# A novel *SOX10* variant in a Japanese girl with Waardenburg syndrome type 4C and Kallmann syndrome

**DOI:** 10.1038/s41439-020-00118-6

**Published:** 2020-09-28

**Authors:** Junpei Hamada, Fumihiro Ochi, Yuka Sei, Koji Takemoto, Hiroki Hirai, Misa Honda, Hironori Shibata, Tomonobu Hasegawa, Mariko Eguchi

**Affiliations:** 1 Department of Pediatrics, Ehime University Graduate School of Medicine, Ehime, Japan; 2 Department of Pediatrics, Ehime Prefectural Niihama Hospital, Ehime, Japan; 3 Department of Pediatrics, Ehime Prefectural Central Hospital, Ehime, Japan; 4 Department of Pediatrics, Keio University School of Medicine, Tokyo, Japan

**Keywords:** Hypogonadism, Disease genetics

## Abstract

We report the first case of Waardenburg syndrome type 4C and Kallmann syndrome in the same person. The patient, a Japanese girl, presented with bilateral iris depigmentation, bilateral sensorineural hearing loss, Hirschsprung disease, hypogonadotropic hypogonadism, and anosmia. We identified a novel *SOX10* variant, c.124delC, p.Leu42Cysfs*67.

Waardenburg syndrome (WS), a rare autosomal dominant disorder, is characterized by sensorineural hearing loss and pigmentation abnormalities of the hair, irises and skin^1^. WS is classified into four subtypes, WS1, WS2, WS3, and WS4. WS4 is defined as a complication of Hirschsprung disease. WS4 is genetically heterogeneous, with three causative genes identified^2^. WS, type 4C (WS4C, OMIM#613266) is caused by heterozygous mutation in the *SOX10* gene, whereas WS, type 4A (WS4A, OMIM#277580) and WS, type 4B (WS4B, OMIM#613265) are caused by mutation of *EDNRB* and *EDN3*, respectively. *SOX10* belongs to the SOX family, the members of which have a high mobility group (HMG) DNA binding domain. SOX10 plays a major role in the development and migration of neural crest cells, which can differentiate into melanocytes, olfactory ensheathing cells, and enteric ganglia neurons^1–5^.

Kallmann syndrome (KS) is a clinically and genetically heterogeneous disorder defined by hypogonadotropic hypogonadism (HH) and olfactory dysfunction. KS is occasionally associated with hearing loss, which occurs in approximately 5% of patients^6^. Several genetic variants have been linked to the etiology of KS with hearing loss, including *KAL1*, *FGFR1*, *FGF8*, *IL17RD*, and *CHD7*^7–10^. Recently, variants in *SOX10* have been identified in a few KS patients with hearing loss^11^.

Here, we report for the first time a case with both WS4C and KS due to a novel *SOX10* variant. The proband was referred to our outpatient clinic at 14.9 years of age for lack of pubertal development. She was born to nonconsanguineous Japanese parents at 39 weeks and 6 days of gestation. The pregnancy and delivery were uncomplicated. Her birth weight and length were 2890 g (−0.6 SD) and 47.0 cm (−1.3 SD), respectively. Shortly after birth, bilateral iris depigmentation was noted (Fig. 1a, b). At 24 h of life, she exhibited abdominal distention, vomiting, and delayed meconium excretion. The intraoperative findings showed caliber changes. The rectal mucosal biopsy sample was positive for acetylcholinesterase staining, which confirmed the diagnosis of Hirschsprung disease. The newborn hearing screening test produced a ‘refer’ result, and auditory brainstem response (ABR) revealed congenital bilateral sensorineural hearing loss. Thus, she was clinically diagnosed with WS4. Her mental development was normal. On the first visit at 14.9 years of age, her height was 142.2 cm (−2.79 SD). She showed no pubic hair or breast development (Tanner stage 1). Endocrinological evaluation showed poor gonadotropin response after gonadotropin-releasing hormone stimulation; basal and peak LH values of <0.2 mIU/ml (reference range: 0.4–4.1) and 3.1 mIU/ml (reference range: 8.5–15.5), respectively; basal and peak FSH values of 1.1 mIU/ml (reference range: 4.8–10.4) and 8.5 mIU/ml (reference range: 8.3–20.0), respectively, and a low plasma estradiol level (10.0 pg/ml; reference range, 11.0–172). The secretion of the other pituitary hormones was normal. The chromosome G-banding analysis showed 46,XX. Intravenous olfactometry (alinamin test) induced no response, indicating anosmia. Brain magnetic resonance imaging (MRI) revealed olfactory bulb agenesis but no abnormalities in the hypothalamus or pituitary (Fig. 1c, d). Her parents and older brother had normal height and age-appropriate pubertal development. Finally, this case was diagnosed as having WS4 and KS.

**Fig. 1:**
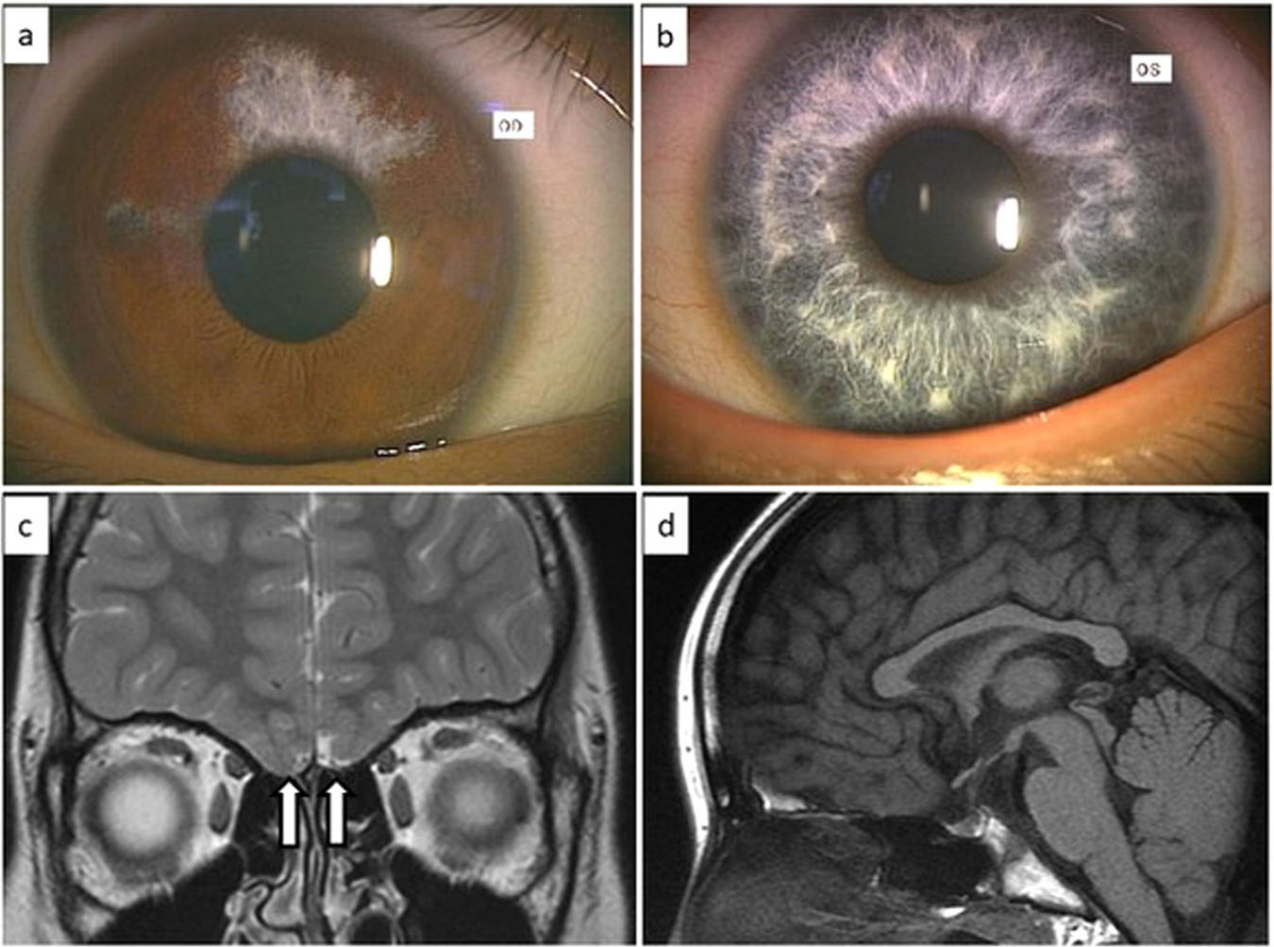
The picture of iris, and MRI findings. **a**, **b** Iris depigmentation. **a** Right (partial depigmentation). **b** Left (complete depigmentation). **c**, **d** T2-weighted brain magnetic resonance imaging. **c** White arrows show olfactory bulb agenesis. **d** No abnormalities were noted in the hypothalamus and pituitary.

We received approval for the genetic study from the Ethics Committee of Ehime University Graduate School of Medicine and the Ethics Committee of Keio University School of Medicine. After obtaining written informed consent from her parents, we extracted genomic DNA from peripheral blood samples from the patient. We analyzed major causative genes and possible causative genes for KS and/or HH, i.e., *AXL*, *CCDC141*, *CHD7*, *DMXL2*, *DUSP6*, *FEZF1*, *FGF8*, *FGF17*, *FGFR1*, *FLRT3*, *FSHB*, *GH1*, *GHR*, *GHRH*, *GHRHR*, *GHSR*, *GLI2*, *GNRH1*, *GNRHR*, *GPR161*, *HESX1*, *HS6ST1*, *IGF1*, *IGF1R*, *IL17RD*, *KAL1*, *KISS1*, *KISS1R*, *LHB*, *LHX3*, *LHX4*, *LEP*, *LEPR*, *MKRN3*, *NELF*, *OTUD4*, *OTX2*, *PAX6*, *PCSK1*, *PGM1*, *PNPLA6*, *POLR3A*, *POLR3B*, *POU1F1*, *PROK2*, *PROKR2*, *PROP1*, *RNF216*, *SEMA3A*, *SEMA7A*, *SIX3*, *SIX6*, *SOX2*, *SOX10*, *SPRY4*, *STAT5B*, *STUB1*, *TAC3*, *TACR3*, *TBX19*, and *TUBB3* using next generation sequencing on a MiSeq instrument (Illumina, San Diego, CA, USA), according to the SureSelect protocol (Agilent Technologies, Santa Clara, CA, USA). We identified a novel heterozygous variant, c.124delC, p.Leu42Cysfs*67, in exon 3 in *SOX10* (Fig. 2). This variant was not found in the Human Genetic Variation Database (http://www.hgvd.genome.med.kyoto-u.ac.jp/), the gnomAD browser (https://gnomad.broadinstitute.org/), or the 1000 Genome Browser (https://www.ncbi.nlm.nih.gov/variation/tools/1000genomes/). No further pathogenic variant was identified in the other tested genes.

**Fig. 2:**
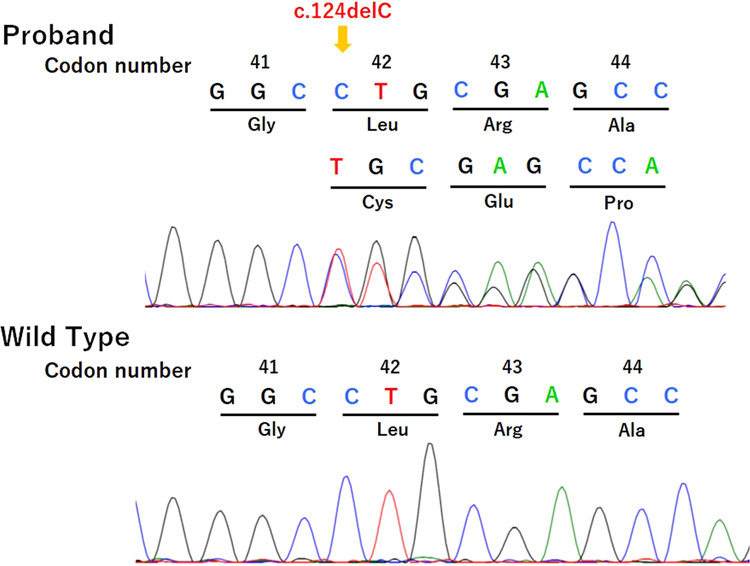
Partial chromatograms of *SOX10*. The upper panel shows a chromatogram of the proband, who had a heterozygous variant, c.124delC, p.Leu42Cysfs*67, which is denoted by an arrow. The lower panel shows a chromatogram of a wild-type sample.

This is the first case of a patient with both WS4C and KS. We, however, cannot deny the possibility that previous reports of WS4C have not been well investigated for gonadal function. When the patient is diagnosed with WS4C in infancy, it is impossible to determine whether the patient has hypogonadism unless he or she is followed until pubertal age.

A novel heterozygous variant, c.124delC, p.Leu42Cysfs*67, in *SOX10* was responsible for the WS4C and KS in this case. This frameshift mutation results in a stop codon, theoretically leading to nonsense-mediated messenger RNA decay, and probably causes haploinsufficiency of *SOX10*. The nonsense mutation p.Glu189* in exon 4 in *SOX10* has been reported in a patient with WS4^12^ and in a patient with KS, WS1, and hyperthyroidism^13^. Notably, Inoue et al.^14^ reported that the p.Glu189* and p.Tyr207* mutations in exon 4 in *SOX10* showed nonsense-mediated messenger RNA decay. Izumi et al.^15^ determined that the p.Pro169fs*117 mutation in *SOX10*, which was found in a female patient diagnosed with KS and WS2, was haploinsufficient by an in vitro assay. Amato et al.^16^ reported a large heterozygous deletion involving the entire coding region of *SOX10* in a woman with KS and bilateral sensorineural hearing loss. These findings together with the present case suggest that *SOX10* haploinsufficiency has wide clinical variation.

In summary, we identified a novel *SOX10* variant in a Japanese girl first diagnosed with WS4C and KS. Patients with WS4C due to a *SOX10* variant should be examined and followed for clinical features of KS.

## Data Availability

The relevant data from this Data Report are hosted at the Human Genome Variation Database at 10.6084/m9.figshare.hgv.2906.

## References

[1] Pingault V (2010). Review and update of mutations causing Waardenburg syndrome. Hum. Mutat..

[2] Chaoui A (2011). Identification and functional analysis of SOX10 missense mutations in different subtypes of Waardenburg syndrome. Hum. Mutat..

[3] Hou L, Arnheiter H, Pavan WJ (2006). Interspecies difference in the regulation of melanocyte development by SOX10 and MITF. Proc. Natl Acad. Sci. USA.

[4] Barraud P, St John JA, Stolt CC, Wegner M, Baker CV (2013). Olfactory ensheathing glia are required for embryonic olfactory axon targeting and the migration of gonadotropin-releasing hormone neurons. Biol. Open.

[5] Kuhlbrodt K, Herbarth B, Sock E, Hermans-Borgmeyer I, Wegner M (1998). SOX10, a novel transcriptional modulator in glial cells. J. Neurosci..

[6] Quinton R (2001). Idiopathic gonadotrophin deficiency: genetic questions addressed through phenotypic characterization. Clin. Endocrinol. (Oxf.).

[7] Marlin S (2013). Discovery of a large deletion of KAL1 in 2 deaf brothers. Otol. Neurotol..

[8] Costa-Barbosa FA (2013). Prioritizing genetic testing in patients with Kallmann syndrome using clinical phenotypes. J. Clin. Endocrinol. Metab..

[9] Suzuki E (2014). De novo frameshift mutation in fibroblast growth factor 8 in a male patient with gonadotropin deficiency. Horm. Res Paediatr..

[10] Miraoui H (2013). Mutations in FGF17, IL17RD, DUSP6, SPRY4, and FLRT3 are identified in individuals with congenital hypogonadotropic hypogonadism. Am. J. Hum. Genet..

[11] Pingault V (2013). Loss-of-function mutations in SOX10 cause Kallmann syndrome with deafness. Am. J. Hum. Genet..

[12] Pingault V (1998). SOX10 mutations in patients with Waardenburg-Hirschsprung disease. Nat. Genet..

[13] Wang F, Zhao S, Xie Y, Yang W, Mo Z (2018). De novo SOX10 nonsense mutation in a patient with Kallmann syndrome, deafness, iris hypopigmentation, and hyperthyroidism. Ann. Clin. Lab Sci..

[14] Inoue K (2004). Molecular mechanism for distinct neurological phenotypes conveyed by allelic truncating mutations. Nat. Genet..

[15] Izumi Y (2015). Hypogonadotropic hypogonadism in a female patient previously diagnosed as having Waardenburg syndrome due to a SOX10 mutation. Endocrine.

[16] Amato LGL (2019). New genetic findings in a large cohort of congenital hypogonadotropic hypogonadism. Eur. J. Endocrinol..

